# Tobacco Product Users Among School-Going Adolescents in Malaysia: Prevalence and Associated Factors

**DOI:** 10.21315/mjms-11-2024-907

**Published:** 2025-04-30

**Authors:** Kavita Jetly, Aniza Ismail, Noraryana Hassan, Azmawati Mohammed Nawi

**Affiliations:** 1 Herbal Medicine Research Centre, Institute for Medical Research, National Institutes of Health, Setia Alam, Selangor, Malaysia; 2 Department of Public Health Medicine, Faculty of Medicine, Universiti Kebangsaan Malaysia, Cheras, Kuala Lumpur, Malaysia; 3 Tobacco Control Unit, Disease Control Division (NCD), Ministry of Health Malaysia, Putrajaya, Malaysia

**Keywords:** smoking, vaping, adolescents, tobacco products

## Abstract

**Background:**

Tobacco use, usually initiated during adolescence, represents a significant public health problem worldwide. This study aimed to determine the prevalence of current users and ever users of tobacco products among adolescents and their associated factors.

**Methods:**

This was a cross-sectional study which was conducted among Form 1, Form 2 and Form 4 students in six secondary schools in the Selangor state of Malaysia. A pre-tested and validated self-administered questionnaire was used.

**Results:**

A total of 386 adolescents agreed to participate and fulfilled the inclusion criteria, giving a response rate of 89.4%. The prevalence of current users and ever users of all tobacco products was 9.8% and 26.2%, respectively. Vape or e-cigarettes recorded the highest prevalence of current users (8.0%) and ever users (17.4%), followed by cigarette smoking (current users: 3.1%; ever users: 14.0%) and snuff (current users: 2.1%; ever users: 9.6%). Tobacco products that recorded a lower prevalence of current and ever users included cigars, chewing tobacco, and pipe smoking (0.3% to 0.8%). The significant factors for current and ever users of tobacco products usage were Malay ethnicity, males, fathers smoking, peers who smoke, and interaction between gender and ethnicity.

**Conclusion:**

In conclusion, about 1 in 10 adolescents were current smokers, and 1 in 4 were ever-smokers. The most popular method of tobacco consumption (current and ever usage) was vaping. Personal, parental, and peer influences were key predictors of smoking, highlighting the need to address them in anti-smoking programmes.

## Introduction

According to the World Health Organization (WHO), around 8 million people die from tobacco use. More than 7 million deaths are due to direct tobacco use, while 1.2 million die due to exposure to second-hand smoke (1). Tobacco use is defined as tobacco leaf placed in tobacco products. In general, tobacco usage can be divided into smoked and smokeless tobacco products. Smoked tobacco products include manufactured cigarettes, hand-rolled cigarettes, roll-your-own with cigarette paper, cigars, pipe smoking, and shisha (2). The most common form of smoked tobacco products worldwide is cigarette smoking (2, 3). The usage of smokeless tobacco involves electronic cigarettes, chewing tobacco, and snuff (4). Smokeless tobacco is not burned, contains nicotine, and attracts adolescents due to the presence of flavours (5). Despite how tobacco is used, its usage causes addiction and detrimental health effects (6, 7).

Tobacco product usage and dependence usually start during adolescence (8, 9). Adolescence is a critical age when individuals start to smoke (10). During this period, adolescents’ knowledge of the effects of smoking appears to be poor (11, 12). In a study by Xu et al. (11), less than 20% of adolescents know that smoking could cause heart disease, peptic ulcers, and cerebral stroke (13). Besides that, adolescents are influenced by “feel-good syndrome,” wanting to look cool, and inquisitiveness, making them vulnerable to smoking (14). Smoking at an earlier age is a public health problem as it causes individuals to become dependent on nicotine faster (15). Adolescents are highly affected by nicotine addiction, which affects their brain development (9). Those addicted to nicotine have a higher risk of consuming tobacco lifelong. The prevalence of tobacco usage among adolescents aged 13–15 years worldwide was substantial (16). The current tobacco usage was 17.9% in boys and 11.5% in girls. The most recent global prevalence of cigarette smoking was 11.3% in boys and 6.1% in girls. The prevalence of current cigarette smoking increased in 21 (15.3%) out of 137 countries. In Malaysia, smoking among adolescents is also a substantial problem. The prevalence of tobacco smokers among youth (13 to 15 years) also only showed a minimal decrease from 2011 to 2016 from 20.2% to 15.9%, respectively (2, 17). More than half of the individuals start smoking before the age of 18 (18). Serial 10-year data from the National Health and Morbidity Survey NHMS for the years 1996 and 2006 (19, 20) also showed a lowering mean age of smoking initiation, from 19.9 to 18.6. According to the Tobacco and E-Cigarette Survey among Malaysian Adolescents (TECMA) 2016 (2) and NHMS (18), the most common form of tobacco usage among adolescents was cigarettes, followed by vaping or e-cigarettes. Many factors can predispose adolescents to smoking (21, 22). These include personal factors such as gender (23, 24), family factors which include parent smoking (25), parent socioeconomic status (26, 27) and social factors such as peer smoking (28). Although multiple programmes have been conducted to reduce smoking prevalence in Malaysia, smoking prevalence has not been reduced among adolescents and a younger age of smoking initiation instead is observed (29). Hence, it is crucial to determine the recent prevalence and factors of tobacco use among adolescents. These findings help support the End Game of Tobacco proposed by the WHO to produce a tobacco-free nation by 2040 and also support Malaysia’s proposed initiative to restrict tobacco product sales to those born after 2005 to create a smoke-free generation (30, 31).

Hence, this study aimed to determine the prevalence of current users and ever users of tobacco products among adolescents and their associated factors. Most of the studies conducted in Malaysia evaluated the prevalence or factors of only a few types of tobacco product usage among adolescents (32–34). However, this study captures the extensive use of tobacco products (smoking and smokeless), including vape or e-cigarettes, cigarettes, snuff, traditional hand-rolled cigarettes, “roll-your-own” with cigarette paper, shisha, cigars, chewing tobacco, and pipe smoking, affecting practices that are tailored to local use. Moreover, their current and ever-use status was evaluated, offering a more comprehensive evaluation than previous local studies. This provided evidence for public health actions and priorities. Policymakers can implement targeted interventions for commonly used tobacco products through stricter regulations, educational campaigns, and strategic resource allocation.

## Methods

### Study Design and Location

This was a cross-sectional study which was conducted in six secondary schools in the Selangor state of Malaysia. Coeducational schools, Malaysian government schools and principals of schools who agreed to take part in this study were included. The schools that were excluded included vocational schools, special education schools, international schools, vernacular schools, sports schools, boarding schools, and private schools.

### Study Population and Sample

This study was conducted among Form 1, Form 2, and Form 4 secondary (aged 12 to 16) students and schooling adolescents from February to July 2021. Malaysian adolescents, adolescents and parents who agreed to participate in this study and those who were able to understand the written and spoken Malay language were included. Those who were absent from school on the day of the survey were excluded.

Sample size calculation was done using StatCalc Epi Info 7.2.4.0. A previous study showed that 28.6% of Malaysian adolescents were ever tobacco users (smokeless and smoked tobacco products) (2), and 20.9% of Malaysian adolescents were current tobacco users (smokeless and smoked) (18). Using a margin of error of 5%, an expected frequency of 28.6%, and a confidence interval of 95%, the calculated sample size was 314 (using the frequency that generated the largest sample size). With a dropout rate of 30%, a total of 448 adolescents were required for this study.

A multi-stage sampling technique was applied to obtain a representative sample. Each district education office constitutes a stratum, and the schools in Selangor’s state were stratified according to their corresponding district education office (35). Out of 10 district education offices, six district education offices were chosen using simple random sampling (SRS). Different district education offices were chosen to increase the generalizability of results, and more than half of the district education offices in Selangor were chosen for this study. Subsequently, the SRS of six secondary schools (one in each district education office) was performed to ensure representation of each school from each district education office.

Next, the SRS of classrooms was conducted to select students from each school. Two classrooms were randomly selected from each form at each school. Two classrooms were chosen since some schools only had about four classrooms for each form. Hence, at least half of these classrooms were randomly selected. Besides, due to COVID-19 and various restrictions imposed, we were unable to sample all classrooms for this study. Proportionate sampling was conducted to determine the number of adolescents chosen from each classroom within each form based on the total student population in each randomly selected classroom. SRS was conducted to select adolescents from each of these classrooms based on the list of students given by the teacher. A total of 143 adolescents from Form 1, 157 from Form 2, and 148 from Form 4 were sampled. Weights were not applied to each stratum since proportionate sampling had been performed earlier (34). Studies have also highlighted that the impact of weighting is very limited and should be used cautiously (36–39).

### Study Tool

A self-administered questionnaire in the Malay language was used in this study. Since the Malay language is the official language for the learning process in school, adolescents were expected to be more well-versed in this language. Besides, using more than one language during data collection can also cause bias (40).

This questionnaire consists of two parts. Part A measures personal factors such as age, gender, ethnicity, and smoking status. Smoking status included “current” or “ever users” of tobacco products (cigarettes, shisha, traditional hand-rolled cigarettes, roll-your-own with cigarette paper, cigar, pipe smoking, vape or e-cigarettes, chewing tobacco, and snuff), age, and first tried a cigarette (for current and ever users of cigarettes). Current users of cigarettes were also asked about the number of cigarettes smoked per day in the past 30 days (2) if they intend to stop cigarette smoking now, and if they tried to stop cigarette smoking in the past 12 months. Ever users were defined as the usage of tobacco products at least once in a lifetime (2, 41). Part B measures social factors (peer smoking) and family factors (parental smoking and parental education).

Questions for smoking status were obtained from TECMA 2016 (a national survey in Malaysia) (2). This questionnaire was developed based on input from experts and researchers in the field of tobacco. It was also adopted by the WHO and the Global Youth Tobacco Survey (GYTS). Besides that, it was available in English and Malay and had been pre-tested and validated. Next, the questionnaire went through a content validity assessment by three field experts in the tobacco field and subsequent pre-testing.

### Data Analysis

Data analysis was performed via IBM SPSS 26.0. Descriptive analysis was used to summarise univariate analysis. Continuous variables (e.g., age) were reported as mean ± SD. All categorical data (e.g., ethnicity) was expressed in frequency and percentages.

Binary logistic regression was performed to determine factors (personal, family, and social characteristics) associated with current or ever-use of tobacco products for multivariable logistic regression at *P* < 0.25 (42). Then, multivariable logistic regression analysis was conducted to determine factors significantly associated with outcome variables. Significance was identified at *P*-values less than 0.05 and the adjusted odds ratio (AOR) at a 95% confidence interval (CI). A few assumptions were tested. The variance-inflated factor (VIF) was used to detect multi-collinearity. A value of VIF > 10 detects multi-collinearity (43, 44). Cook outliers were also determined, and the cut-off value for an influential outlier was above 1.00 (45). Subsequently, the model fit will be tested via the Hosmer and Lemeshow test, and a non-significant test (*P* < 0.05) indicates that the predicted risk of the model is consistent with the actual risk (46). Model fit will also be assessed via a classification table (above 70% indicates a good model) and an area under the curve (above 70% indicates a good fit) (47). Two-way interactions between all independent variables in the final model were also conducted.

## Results

The following describes the results obtained in this study.

### Response Rate

A total of 448 adolescents were sampled; however, some were unavailable on survey day. As a result, 432 adolescents were approached, of whom 386 agreed to participate and fulfilled the inclusion criteria, yielding a response rate of 89.4%.

### Adolescents’ Personal, Family and Social Characteristics

Table 1 shows the personal, family, and social characteristics of adolescents participating in this study. The mean age of adolescents was 15.12 (SD = 1.27). The majority of the adolescents were of Malay ethnicity (75.6%) and male (56.5%). Most of the fathers of adolescents smoked (33.9%), and most had no tertiary education (55.2%). On the other hand, only 7 (1.8%) mothers smoked, and most were also not tertiary educated (54.1%). About 69.7% of adolescents had peers who did not smoke.

### Prevalence of Current and Ever Users of Tobacco Products Among Adolescents and Characteristics of Cigarette Smokers

The prevalence of current users of all tobacco products among adolescents was 9.8%, and the prevalence of ever users was 26.2% (Table 2). Vape or e-cigarettes record the highest prevalence of current users (8.0%) and ever users (17.4%), followed by cigarette smoking (current users: 3.1%; ever users: 14.0%) and snuff (current users: 2.1%; ever users: 9.6%). Tobacco products that recorded a lower prevalence of current and ever users included cigars, chewing tobacco, and pipe smoking, where the prevalence of current and ever users ranged from 0.3% to 0.8%.

The mean age for first smoking cigarettes among adolescents in this study was 12.41 years old (SD = 2.84). The youngest age of cigarette smoking initiation was 7 years old, which was recorded in three adolescents (5.6%). One-third of smokers smoked less than one cigarette in the past 30 days 4 (33.4%), one-third smoked one cigarette per day 4 (33.4%), and one-third smoked more than one cigarette per day 4 (33.4%). However, most (91.2%) intended to stop smoking at the time of the survey, and 11 (91.2%) tried to stop smoking in the past 12 months.

### Current and Ever Users Stratified by Personal, Family and Social Characteristics

Table 3 shows the current and ever users of tobacco products. The majority of both groups were of male gender (current users: 86.8%; ever users: 78.2%) and Malay ethnicity (current users: 89.5%; ever users: 87.1%). Regarding family characteristics, most of the current and ever users had a father smoking (current users: 65.8%; ever users: 54.5%), a father without tertiary education (current users: 57.9%; ever users: 60.4%), and a mother without tertiary education (current users: 55.3%; ever users:56.4%). In terms of social characteristics, a significant proportion of current and ever users had peer smoking (current users: 73.7%; ever users: 57.4%).

### Factors Associated with Current and Ever Users of All Tobacco Products

The results of the univariate and multivariate analysis of personal, family, and social characteristics associated with current and ever users are shown in Tables 4 and 5, respectively. In a simple logistic regression analysis, being male, having Malay ethnicity, having a father or mother who smokes, and having peers who smoke were significantly associated with current tobacco use. For ever users, these factors—male gender, Malay ethnicity, father smoking, and peer smoking were significantly associated with ever tobacco use. Variables with *P* < 0.25 were included in multiple logistic regression analysis (43).

In multiple logistic regression analysis, male gender, Malay ethnicity, father smoking, peer smoking, and the interaction between gender and ethnicity were found to be significant factors for current users of all tobacco products. Males had 3.69 times higher odds of being a current smoker in comparison to females (AOR: 3.69, 95% CI: 2.06–6.59, *P* < 0.001), while Malays had 2.88 times higher odds of being a current user than non-Malays (AOR: 2.88, 95% CI: 1.42–5.83, *P* = 0.003). Having a father who smokes increased the odds of being a current smoker by 3.33 (AOR: 3.33, 95% CI: 1.96–5.65, *P* = < 0.001) compared to non-smoking fathers and having a peer who smokes increased the odds by 3.70 times compared to peers who do not smoke (AOR: 3.70, 95% CI: 2.18–6.28, *P* = < 0.001). Additionally, the interaction between Malay ethnicity and male gender was associated with 15.77 times higher odds of being a current smoker (AOR: 15.77, 95% CI: 1.38–180.04, *P* = 0.026).

Male gender, Malay ethnicity, father smoking, father education, peer smoking, and the interaction between gender and ethnicity were found to be significant factors for ever users of all tobacco products. Males had 4.11 times higher odds of being an ever-smoker compared to females (AOR: 4.11, 95% CI: 2.25–7.51, *P* < 0.001), while Malays had 2.88 times higher odds of being an ever-smoker than non-Malays (AOR: 2.88, 95% CI: 1.41–5.87, *P* = 0.004). The likelihood of being an ever-smoker was 3.10 times higher among individuals with a smoking father compared to those with a non-smoking father (AOR: 3.10, 95% CI: 1.82–5.29, *P* < 0.001). Additionally, having a father with no tertiary education increased the odds of 1.93 ever being a smoker compared to fathers with no tertiary education (AOR: 1.93, 95% CI: 1.11–3.35, *P* = 0.020). Besides, having a peer who smokes increased the odds by 3.71 times in comparison to peers who do not smoke (AOR: 3.71, 95% CI: 2.17–6.33, *P* < 0.001). Notably, the interaction between Malay ethnicity and male gender was significantly associated with 9.00 times higher odds of being an ever-smoker (AOR: 9.00, 95% CI: 2.15–37.60, *P* = 0.003).

## Discussion

The prevalence of current users of tobacco products among adolescents obtained in this study was 9.8% (95% CI: 6.9–12.8), and ever users were 26.2% (95% CI: 21.8–30.6). Current users ranged from 2.4% to 30.9%, and ever users were 6.4% to 48.8% (48–59) in neighbouring countries. The prevalence of current and ever users of tobacco products in our study was relatively high compared to national studies in many neighbouring countries. Our study reported the prevalence of current and ever-user users in one state and a few schools compared to all these national studies in neighbouring countries, which were extensively studied in many states and schools. Geographical factors, cultural factors, tobacco control policies and socioeconomic differences between countries can plausibly account for the different prevalence of tobacco product users between countries (60– 62).

The tobacco product with the highest prevalence of current and ever users in this study was vaping or e-cigarettes (current users 8.0%, and ever users 17.4%). Similar to TECMA (2), NHMS (18), and studies worldwide, vaping or e-cigarettes are one of the most common current methods of tobacco consumption (2, 18, 63). Vapes or e-cigarettes have undoubtedly increased in popularity among adolescents in recent years (64–66). This is due to the product appeal of vapes or e-cigarettes with multiple tastes and smells, which attract youth’s curiosity and feelings of experimenting (65, 67, 68). Other methods of tobacco usage that reported the lowest prevalence of current and past usage among adolescents in this study were cigars, chewing tobacco, and pipe smoking. TECMA (2) also noted the similar unpopular usage of these tobacco products. These tobacco products do not appeal to the adolescents of our country. There is reduced popularity of these products, and they are perceived as old-fashioned and not trendy (65).

This study’s mean age of cigarette smoking initiation was 12.41 years old. The age was within a similar range, as reported in a systematic review conducted in Asia (27). Besides that, local research also noted similar findings. One study reported that almost two-thirds of smokers started smoking before they were 14, and another study reported a mean age for smoking of 11.79 years old (32, 33). The young age of smoking initiation among adolescents is because of the incapability of making a rational judgement at this age (69).

Besides that, some parents have rated their adolescents between the ages of 12 and 14 as more disobedient and unable to fully control their emotional urges (70). Neurodevelopmental studies confirm these findings (71). However, the prefrontal input to the subcortical circuitry rises during development to adulthood, which improves control of emotional responses. Besides that, neuroendocrine changes during puberty are associated with increased testosterone, which is responsible for sensation seeking and exploring risky behaviours, including smoking (72). In addition to that, at this young age, adolescents have greater pleasure in substance use, lack concern about the outcomes of smoking in the future, and are more vulnerable to peer pressure (73). Piaget’s cognitive theory can also provide a plausible explanation for adolescents’ smoking behaviour (74).

The significant risk factors for current smokers were being male, Malay, a father smoking, peer smoking, and the interaction between gender and ethnicity. For ever-smokers, male, Malay, father smoking, peer smoking, father education, and the interaction between gender and ethnicity pose a risk factor for smoking.

Previous studies have shown that most of the current and ever users were males (75, 76). Accepting the male gender as a social norm and smoking as an image of masculinity contributes to the above significant findings among both the above groups (77). Adolescents have also perceived males who smoke as having positive characteristics such as strength and maturity (78). The stigma of smoking due to cultural reasons may be more strongly inflicted on female smokers than on male smokers, which can account for gender differences in smoking habits, especially in the Asian population (79, 80). Additionally, females are shown to be more aware of the consequences of smoking and more concerned regarding their health and the health of others (81).

Besides, most adolescents obtain tobacco from their friends, and peer pressure is a key predictor of both current and ever smoking (65, 82). Adolescents tend to have a strong bond and act similarly with their friends to gain recognition, be accepted in a group and avoid being ridiculed (78). Norms of parental and peer smoking are also influential for current and ever smoking among adolescents (28, 82). Parents are viewed as role models, and watching them smoke can act as an enabler for adolescents to smoke, as they will perceive smoking as acceptable, normative, familiar and not prohibited (78, 82). Generally, social norms (parent or peer smoking) may influence smoking behaviour by giving cues or opportunities to be involved in that behaviour (83).

In addition, socioeconomic disadvantage (measured by parental education in this study) has an increased risk of current and ever smoking among adolescents (82, 84). Adolescents with low-educated parents may not be able to provide better life opportunities, such as enrollment in better-quality schools that enforce stronger bans and restrictions on smoking (85). Besides, adolescents from socioeconomically disadvantaged families tend to feel the extreme pressures of life and tend to seek immediate gratification from smoking (86).

Studies have shown that certain ethnic groups are more susceptible to being smokers and current smokers (82, 87). In our study, Malay ethnicity was significantly associated with current and ever smoking, similar to local studies (88, 89). Studies have shown there are factors which link smoking to genetic ancestry (90). The factors that contribute to the interaction between gender and ethnicity, which significantly increases the odds of smoking among current and ever-smokers, can be explored from multiple perspectives, including social and biological determinants.

There were a few strengths of this study. This includes the usage of probability sampling with an adequate sample size and a high response rate. Besides that, the use of a validated questionnaire increased the validity and reliability of the results obtained. In addition, a vast range of types of current and ever users smoked and smokeless (vape or e-cigarettes, cigarettes, snuff, traditional hand-rolled cigarettes, “roll-your-own” with cigarette paper, shisha, cigar, chewing tobacco, and pipe) was evaluated in this study. Most of the local studies conducted in Malaysia evaluated only a few types of tobacco product usage among adolescents (33, 91).

Despite the strengths mentioned above, a few limitations were identified. Self-reported measures were used to determine smoking status in this study, which might have been subjected to reporting and social desirability bias. However, one study reported that self-reported smoking status is congruent with exhaled carbon monoxide if confidentiality is maintained (23). A cross-sectional design was used instead of a longitudinal and experimental study, which limits causality. However, the study findings provide insight into aspects that can be further explored. Finally, since the data collection was done during the COVID-19 pandemic, it might have changed some behaviours and associations in this study.

## Conclusion

In conclusion, about 1 in 10 adolescents were current smokers, and 1 in 4 were ever-smokers. The most popular method of tobacco consumption (current and ever usage) was vaping. Personal, parental and peer influences were key predictors of smoking, highlighting the need to address them in anti-smoking and health education programmes. Future studies can address other predictors, such as life stressors and marketing and advertising factors that can influence smoking among adolescents.

## Figures and Tables

**Table 1:** Adolescent’s personal, family and social characteristics, *n* = 386

Personal, family and social characteristics	*N* (%)
Personal characteristics	
Age, mean (SD)	15.12 (1.27)
Ethnicity	
Malay	292 (75.6)
Chinese	33 (8.5)
Indian	47 (12.2)
Others^a^	14 (3.7)
Gender	
Male	218 (56.5)
Female	168 (43.5)
Family characteristics	
Parent smoking	
Father smoking	131 (33.9)
Father not smoking	255 (66.1)
Mother smoking	7 (1.8)
Mother not smoking	379 (98.2)
Either or both parents smoking	131 (33.9)
Neither parent smoke	255 (66.1)
Father’s education	
No tertiary education	213 (55.2)
Tertiary	173 (44.8)
Mother’s education	
No tertiary education	209 (54.1)
Tertiary education	177 (45.9)
Social characteristics	
Peer smoking	
Yes	117 (30.3)
No	269 (69.7)

Notes:

a Others include Malaysian Indonesian, *n* = 7 (50.0%); Iban, *n* = 3 (21.4%); Dayak Bidayuh, *n* = 1 (7.1%); Kenyah, *n* = 1 (7.1%); Rawa, *n* = 1 (7.1%); Suluk, *n* = 1 (7.1%)

**Table 2:** Prevalence of current and ever users of tobacco products among adolescents

Tobacco products (smoke and smokeless)	Current users^a^ *n* (%)	Ever users *n* (%)
All tobacco products	38 (9.8)	101 (26.2)
Vape or E-cigarette	31 (8.0)	67 (17.4)
Cigarette	12 (3.1)	54 (14.0)
Snuff	8 (2.1)	37 (9.6)
Traditional Hand-rolled Cigarettes	3 (0.8)	19 (4.9)
“Roll-your-own” with Cigarette paper	1 (0.3)	16 (4.1)
Shisha	5 (1.3)	13 (3.4)
Cigar	1 (0.3)	3 (0.8)
Chewing tobacco	2 (0.5)	2 (0.5)
Pipe Smoking	1 (0.3)	2 (0.5)

Note:

a Usage in the past 30 days

**Table 3:** Current and ever users stratified by personal, family and social characteristics

Variables	Current users		Ever users	

	Yes *n* = 38 (%)	No *n* = 348 (%)	Yes *n* = 101 (%)	No *n* = 285 (%)
Personal characteristics				

Gender				
Male	33 (86.8)	185 (53.2)	79 (78.2)	139 (48.8)
Female	5 (13.2)	163 (46.8)	22 (21.8)	146 (51.2)

Ethnicity				
Malay	34 (89.5)	258 (74.1)	88 (87.1)	204 (71.6)
Non-Malay	4 (10.5)	90 (25.9)	13 (12.9)	81 (28.4)

Family characteristics				

Father smoking status				
Father smoking	25 (65.8)	106 (30.5)	55 (54.5)	76 (26.7)
Father not smoking	13 (34.2)	242 (69.5)	46 (45.5)	209 (73.3)

Mother smoking status				
Mother smoking	4 (10.5)	3 (0.9)	4 (4.0)	3 (1.1)
Mother not smoking	34 (89.5)	345 (99.1)	97 (96.0)	282 (98.8)

Father’s education				
No tertiary education	22 (57.9)	191 (54.9)	61 (60.4)	152 (53.3)
Tertiary	16 (42.1)	157 (45.1)	40 (39.6)	133 (46.7)

Mother’s education				
No tertiary education	21 (55.3)	188 (54.0)	57 (56.4)	152 (53.3)
Tertiary education	17 (44.7)	160 (46.0)	44 (43.6)	133 (46.7)

Social characteristics				

Peer smoking				
Yes	28 (73.7)	89 (25.6)	58 (57.4)	59 (20.7)
No	10 (26.3)	259 (74.4)	43 (42.6)	226 (79.3)

**Table 4:** Univariate analysis of personal, family and social characteristics associated with current and ever users of all tobacco products

Variables	Current users				Ever users			

	Crude OR^a^ (95% CI)	B estimate	Wald statistics	*P*-value	Crude OR^a^ (95% CI)	B estimate	Wald statistics	*P*-value
Personal characteristics								

Gender^a^								
Male	5.82 (2.22–15.25)	1.76	12.82	< 0.001	3.77 (2.23–6.39)	1.33	24.42	< 0.001
Female	1				1			

Ethnicity^a^								
Malay	2.97 (1.02–8.59)	1.09	4.01	0.045	2.69 (1.42–5.08)	0.99	9.26	0.002
Non-Malay	1				1			

Family characteristics								

Father smoking status^a^								
Father smoking	4.39 (2.16–8.91)	1.48	16.77	< 0.001	3.29 (2.05–5.27)	1.19	24.49	< 0.001
Father not smoking	1				1			

Mother smoking status^a^								
Mother smoking	13.53 (2.91–62.97)	2.61	11.02	0.001	3.88 (0.852–17.628)	1.36	3.07	0.080
Mother not smoking	1				1			

Father’s education^a^								
No tertiary education	1.13 (0.57–2.23)	0.122	0.125	0.723	1.33 (0.841–2.12)	0.29	1.50	0.221
Tertiary	1				1			

Mother’s education								
No tertiary education	1.05 (0.54–2.06)	0.05	0.021	0.884	1.13 (0.718–1.79)	0.13	0.29	0.591
Tertiary education	1				1			

Social characteristics								

Peer smoking								
Yes	8.15 (3.81–17.44)	2.10	29.18	< 0.001	5.17 (3.17–8.41)	1.64	43.59	< 0.001
No	1				1			

Note: OR = Odds Ratio

**Table 5:** Multivariate analysis of personal, family and social characteristics associated with current and ever users of all tobacco products

Variables	Current users				Ever users			

	AOR (95% CI)	B estimate	Wald statistics	*P*-value	AOR (95% CI)	B estimate	Wald statistics	*P*-value
Personal characteristics								

Gender^a^								
Male	3.69 (2.06–6.59)	1.31	19.42	< 0.001	4.11 (2.25–7.51)	1.41	21.17	< 0.001
Female	1				1			

Ethnicity^a^								
Malay	2.88 (1.42–5.83)	1.06	8.61	0.003	2.88 (1.41–5.87)	1.06	8.42	0.004
Non-Malay	1				1			

Family characteristics								

Father smoking status^a^								
Father smoking	3.33 (1.96–5.65)	1.20	19.76	< 0.001	3.10 (1.82–5.29)	1.13	17.30	< 0.001
Father not smoking	1				1			

Mother smoking status^a^								
Mother smoking								
Mother not smoking								

Father’s education^a^								
No tertiary education					1.93 (1.11–3.35)	0.66	5.41	0.020
Tertiary					1			

Mother’s education								
No tertiary education								
Tertiary education								

Social characteristics								

Peer smoking								
Yes	3.70 (2.18–6.28)	1.31	23.47	< 0.001	3.71 (2.17–6.33)	1.31	23.06	< 0.001
No	1				1			

Gender × ethnicity (Malay × male)	15.77 (1.38–180.04)	2.76	4.93	0.026	9.00 (2.15–37.60)	2.20	9.07	0.003
Gender × father smoking (male × father smokes)	1.55 (0.21–11.75)	0.44	0.18	0.670	1.91 (0.61–5.99	0.65	1.23	0.267

Gender × father education (male × father no tertiary education)					1.25 (0.35–4.44)	0.22	0.11	0.736

Ethnicity × peer smoking (Malay × peer smokes)	0.83 (0.07–10.23)	–0.19	0.02	0.884	0.730 (0.172–3.11)	–0.32	0.18	0.670

Father education × father smoking (father no tertiary education × father smoking)					0.428 (0.144–1.268)	–0.85	2.35	0.126

Notes: Both backward and forward likelihood ratio (LR) methods yielded similar results. Assumptions tested—Current smokers: no multi-collinearity (variance-inflated factor [VIF] = 1.02–1.10); Hosmer-Lemeshow = 0.932; classification accuracy = 89.6%; predicted probabilities = 0.856; no Cook outliers present. Ever users: no multicollinearity (VIF = 1.02–1.15); Hosmer-Lemeshow = 0.152; classification accuracy = 79.3%; predicted probabilities = 0.82; no Cook outliers present.

## Data Availability

The datasets used and analysed during the current study are available from the corresponding author upon reasonable request.
